# Systems Pharmacology and Molecular Docking Reveals the Mechanisms of Nux Vomica for the Prevention of Myasthenia Gravis

**DOI:** 10.1155/2022/9043822

**Published:** 2022-06-26

**Authors:** Chao Qiu, Qiang Chen, Qun Hou, Guanshu Qi

**Affiliations:** Department of Neurology, The First Affiliated Hospital of Zhejiang Chinese Medical University, Hangzhou 310006, China

## Abstract

**Background:**

Myasthenia gravis (MG) is a rare autoimmune disease with clinical symptoms of fluctuating muscle weakness. Due to the side effects of current therapies, there is an urgent need for a new medication for MG treatment. Nux vomica is a traditional Chinese medicine used in various diseases. However, the mechanism of action of Nux vomica against MG remains unclear.

**Methods:**

Network pharmacology was used to explore the underlying mechanisms of Nux vomica in MG treatment, which was validated using molecular docking and *in vivo* experiments in mice.

**Results:**

Twelve bioactive compounds and 72 targets in Nux vomica were screened. Seventy-nine myasthenia-related targets were obtained from the GENECARD and DisGeNET databases. PPI networks of Nux vomica- and myasthenia-related targets were constructed using Bisogenet, and these two networks were subsequently merged into an intersection to establish a core-target PPI network that consisted of 204 nodes and 4,668 edges. KEGG enrichment analysis indicated that 132 pathways were enriched in 204 core targets. In addition, we obtained 50 docking pairs via molecular docking. *In vivo* experiments revealed that Nux vomica can improve the symptoms of MG.

**Conclusion:**

Nux vomica is involved in the pathogenesis of MG through the “multicomponent-target-pathway” mechanism.

## 1. Introduction

Myasthenia gravis (MG) is an autoantibody-mediated neuromuscular disease that causes fluctuating muscle weakness predominantly in the ocular, bulbar, and proximal limb muscles [1]. The prevalence rate is approximately one in 5,000 worldwide, and the incidence is higher in younger women and older men [2]. Currently, patients with MG are mainly treated with corticosteroids, long-term immunosuppressive drugs, plasma exchange, or thymectomy [3]. However, their clinical application is limited due to their high costs and side effects, such as cardiac rhythm disorders and hypotension [4]. Hence, alternative therapies with higher efficacy and fewer side effects are needed for MG treatment.

Traditional Chinese medicines (TCMs) have received increasing attention because of their high efficacy, safety, and few adverse effects. According to the TCM theory, MG is classified as “Weizheng” and diagnosed as “flaccidity syndrome” and “Qi-deficiency pattern” [5]. Thus, Qi-supplementation therapy is important for MG treatment. An increasing number of studies have shown that TCMs as an alternative or adjuvant of Western medicines could significantly improve the clinical efficacy of MG treatment [6–8]. Jianpiyiqi granules have been shown to remarkably alleviate the clinical symptoms in children with ocular MG accompanied by a decrease in acetylcholine receptor autoantibodies [6]. In addition, the Qiangji Jianli decoction has been shown to alleviate oxidative stress injury and facilitate mitochondrial biogenesis via the AMPK/PGC-1*α*-dependent signaling pathway, which restored muscle energy supply and ameliorated gastrocnemius muscle contractility in experimental autoimmune MG rats [7]. Another well-known TCM treatment for MG, Bu Zhong Yi Qi decoction, has been reported to decrease simulated weightlessness-induced muscle atrophy and promote myogenesis by reducing nuclear receptor corepressor 1 (NCoR1)-associated gene expression [8]. Nux vomica, also called Semen Strychni or Ma qianzi (Chinese: 马钱子), is the seed of Strychnos Nux-vomica *L*, belonging to the genus Strychnos of the family Loganiaceae. Studies have illustrated that Nux vomica has numerous biological activities, such as analgesic, anti-inflammatory, antitumor, and immunomodulatory effects [9]. A previous study found that Nux vomica could alleviate inflammatory response and thereby ameliorates MG in experimental autoimmune MG rats [10]. However, the molecular mechanisms of Nux vomica against MG remain unclear. Therefore, it is meaningful to explore the targets and further clarify the underlying mechanisms of action of Nux vomica on MG treatment based on network pharmacology.

Network pharmacology, first introduced by British pharmacologist Hopkins, has proven to be an effective strategy for revealing the complicated relationship between TCMs and diseases [11, 12]. TCMs exert their therapeutic functions through a multicompound-target-pathway network. Thus, the molecular mechanisms of TCMs can be clarified using network pharmacology systematically and integrally [13]. In this study, we screened the active components of Nux vomica and explored its underlying molecular mechanisms on MG based on network pharmacology. Our study provides a theoretical basis for the future clinical application of Nux vomica for treating MG.

## 2. Materials and Methods

### 2.1. Collection of Therapeutic Targets for MG

Therapeutic targets for MG were obtained from GeneCards [14] (https://www.genecards.org/) and DisGeNET [15] (https://www.disgenet.org/) databases. Candidate targets were collected after the deletion of duplicates.

### 2.2. Collection of Bioactive Ingredients and Therapeutic Targets for Nux Vomica

TCMSP (https://tcmspw.com/tcmsp.php) is an online platform for integrating pharmacokinetics, medicinal chemistry, therapeutic targets, and drug-target-disease networks [16]. Based on the TCMSP platform, bioactive ingredients and targets of Nux vomica were obtained using the screening criteria of oral bioavailability (OB) > 30% and drug-likeness (DL) > 0.18 [17]. In addition, strychnine was considered as a candidate bioactive ingredient based on a previous study. The pharmacokinetics and DL properties of strychnine were further assessed using the online SwissADME database (https://www.swissadme.ch/index.php). The therapeutic targets of strychnine were then predicted using the SwissTargetPrediction online tool (https://swisstargetprediction.ch/).

### 2.3. PPI Network Construction

A PPI network was constructed and visually analyzed using the Cytoscape plugin Bisogenet, which contains the following six PPI databases: the IntAct Molecular Interaction Database (IntAct), Biomolecular Interaction Network Database (BIND), Biological General Repository for Interaction Datasets (BioGRID), Database of Interacting Proteins (DIP), Molecular INTeraction Database (MINT), and Human Protein Reference Database (HPRD) [18]. PPI networks of the Nux vomica-related targets and MG-related targets were constructed using Cytoscape.

### 2.4. Central Network Evaluation

Central network evaluation is considered a primary method for core protein screening in PPI networks. First, the PPI networks of the Nux vomica-related targets and MG-related targets were merged into an intersection. Next, the Cytoscape plugin CytoNCA was applied to assess the intersection from the following six centrality measures: betweenness centrality (BC), closeness centrality (CC), degree centrality (DC), eigenvector centrality (EC), network centrality (NC), and local average connectivity-based method (LAC) [19]. In addition, CytoHubba, a Cytoscape plugin, was used to select the hub genes [20].

### 2.5. GO and KEGG Pathway Enrichment Analyses

Gene ontology (GO) and Kyoto Encyclopaedia of Genes and Genomes (KEGG) pathway enrichment analyses were performed based on the public database GO (https://geneontology.org/) and KEGG (https://www.genome.jp/kegg/).

A hypergeometric distribution model was used to evaluate whether the target gene set was significantly associated with a specific gene ontology or biological pathway using the following formula:

(1) $$\begin{array}{c} P=1-\sum_{i=0}^{k-1}\frac{\left(\begin{array}{c} M\\ i \end{array}\right)\left(\begin{array}{c} N-M\\ n-i \end{array}\right)}{\left(\begin{array}{c} N\\ n \end{array}\right)}. \end{array}$$

Here, *N* represents the total number of genes from the reference terms, *M* represents the number of genes annotated to specific GO terms or pathways, *n* represents the number of identified Nux vomica-target genes, and *k* represents the number of common genes between Nux vomica-target genes and the reference set. The clusterProfiler package in R (4.0) (https://www.bioconductor.org/packages/release/bioc/html/clusterProfiler.html) was adopted to perform the GO and KEGG pathway enrichment analyses with *P* values <0.01, adjusted by the Bonferroni correction.

### 2.6. Molecular Docking Analysis

The zinc database (https://zinc15.docking.org) was used to download two-dimensional (2D) structures of bioactive compounds [21]. AutoDockTools-1.5.6 was used to add nonpolar hydrogen, calculate charges, and set rotatable keys for the structure and save it as a “pdbqt” file [22]. The 3D structures of the hub targets were downloaded from the Protein Data Bank (PDB) database (https://www.rcsb.org/) [23]. The hub targets and the original ligands were separated and the water molecules were removed using PyMol 2.3.0 [24]. Next, the ligand and receptor were imported into AutoDockTools-1.5.6 to add nonpolar hydrogen, calculate charges, and save as a “pdbqt” file. The protein proligand was set as the center of the docking box; if no proligand was present in the target protein, the docking region was identified based on key amino acid residues near the active pocket of the target, as reported in previous studies, or the fpocket [25] online pocket parameter prediction website for analysis. The prepared protein files, ligand files, compound files, and scripts were placed in the appropriate folders, and molecular docking was performed using AutoDock Vina 1.1.2 [26]. Finally, the binding affinity was calculated using AutoDock Vina 1.1.2, and the docking results were visualized using the open-source version PyMol 2.3.0.

### 2.7. Animal Experiments

Female C57BL/6J mice (aged 7–8 weeks) were purchased from the Animal Resources Center (Murdoch, WA, Australia). All mice were housed in a controlled environment under a 12-h light/dark cycle with 21°C and 45–65% humidity and had free access to food and water.

After anaesthesia, mice in the model group were emulsified with 40 *μ*g MuSK in 100 *μ*L phosphate-buffered saline (PBS) and 100 *μ*L complete Freund's adjuvant (CFA; Becton, Dickinson and Company, NJ, USA), which were intraperitoneally injected into the hind foot. The control group was injected with 100 *μ*L of PBS and 100 *μ*L of CFA. The mice in both groups were injected with cyclophosphamide monohydrate (300 mg/kg, dissolved in 0.9% saline to a final concentration of 10 mg/mL; Sigma-Aldrich, MO, USA) within 24 h to suppress immune resistance. The aforementioned operation was repeated after 30 days. The body weight of the mice was measured weekly, and a clinical examination was performed every 2 weeks to determine the model-building cycle. After the MG mouse model was successfully constructed, the mice were randomly divided into five groups (*n* = 8). The mice in the low-, mid-, and high-dose Nux vomica groups were administered low (4 mg/kg), mid (8 mg/kg), and high (16 mg/kg) doses of Nux vomica. The mice in the control and model groups were intragastrically administered equivalent doses of saline. The body weight of the mice was measured weekly, and a clinical examination was performed every 2 weeks. After 30 days, mice were sacrificed by cervical dislocation after injecting sodium pentobarbital (50 mg/kg), and tendon tissues were removed for subsequent experiments.

### 2.8. Clinical Evaluation

For clinical examination, mice were left for 3 min on a flat platform and observed for signs of MG. The clinical muscle weakness was graded as follows: Grade 0.5, the mouse exhibited slightly lower activity than normal after the standard exercise state, and the hanging time was >60 s; Grade 1, the mouse exhibited normal movement, lying prone, staying still, and/or hanging time <60 s; Grade 2, before and after exercise, the mouse was lying prone, staying still, and/or hanging test <30 s; Grade 3, severe weakness, dehydration and paralysis, weight loss >15%, and/or hanging time 0 s; Grade 4, the mouse died. The grip of the mice was observed every 2 weeks. First, the tail of each mouse was gently grabbed and the mouse was suspended above a metal grid. When the mouse grasped the metal grid, the mouse was lifted and the tension was measured 20 times. One end was fixed on the tension device, and the front end of the force measurement device was made into a grid shape. The tail of the mouse was grasped, the front end of the force measuring device was grasped, and the tail of the mouse was pulled forcefully. When the mouse was not strong enough to grasp the front end of the force measuring device, the force measuring device showed the grip force at this time. In addition, clinical progress was assessed using an inverted screening test every 2 weeks. In this test, the mouse was placed in the center of the grid and immediately rotated to the upside-down position, and stably maintained 50 cm above the soft cushion. The time when the mouse fell was recorded and the observation time was limited to 300 s.

### 2.9. Quantitative Real-Time PCR (qRT-PCR) Assay

Total RNA was extracted from the tendon tissues of the mice using TRIzol® Reagent (Ambion™) (Applied Biosystems, MA, USA), and 500 ng of RNA was reverse-transcribed into cDNA using TaqMan® Reverse Transcription Reagents (Thermo Fisher Scientific, MA, USA). The expression of target genes was examined using ABI Prism SDS 7000 (Thermo Fisher Scientific) at 95°C for 1 min, followed by 40 cycles of 95°C for 15 s, and 60°C for 1 min, and then calculated using the 2^−ΔΔCt^ method. Primer sequences for qRT-PCR are listed in Table S1. GAPDH was used as an internal control.

### 2.10. Western Blot Analysis

The predicted target protein expression was detected by western blotting. Briefly, total proteins were extracted from tendon tissue using RIPA lysis buffer (Applied Biosystems, MA, USA). Then, the proteins were separated by sodium dodecyl sulfate-polyacrylamide gel electrophoresis and transferred to a polyvinylidene fluoride membrane (Millipore, MA, USA). The membranes were blocked with 5% skimmed milk and incubated with primary antibodies anti-EGFR, anti-TP53, anti-AKT1, anti-MYC, and anti-GAPDH (1 : 1,000, Abcam, UK) at 4°C overnight. After washing with PBS, the membranes were incubated with a secondary antibody (1 : 1,000, Abcam). Enhanced chemiluminescent reagents (Millipore, MA, USA) were used to detect the protein bands. GAPDH was used as an internal control.

### 2.11. Statistical Analysis

Data are shown as the mean ± standard deviation (SD) and analyzed by one-way ANOVA followed by Tukey's test using SPSS 27.0 software (IBM, IL, USA). Differences were considered significant at *P* < 0.05.

## 3. Results

### 3.1. Nux Vomica Bioactive Compounds-Targets Network

**Figure 1:**
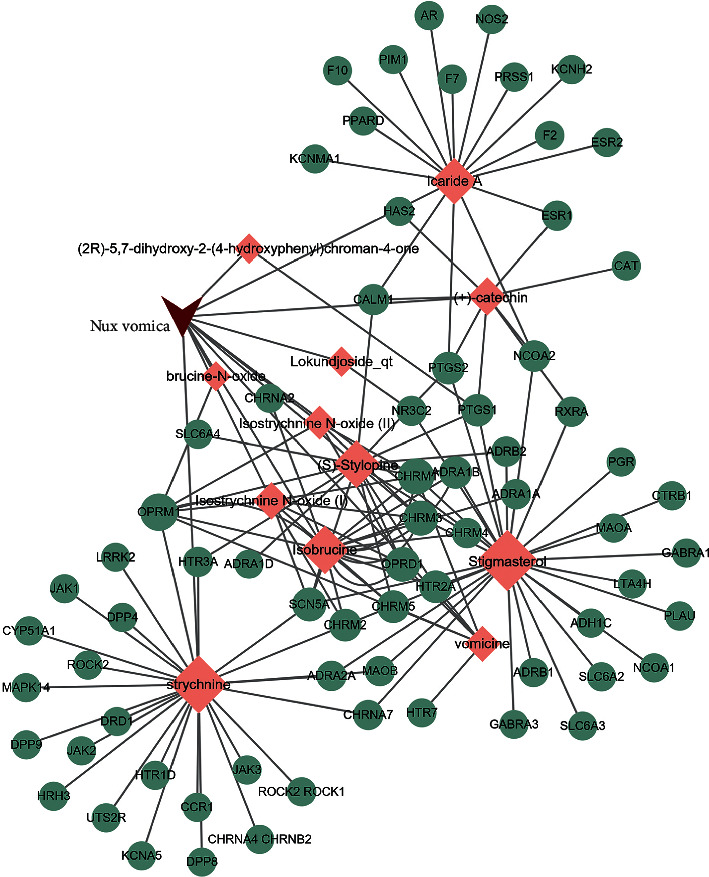
Nux vomica bioactive compounds-targets network. Red inverted triangle represents Nux vomica, pink rhombus represents bioactive compounds of Nux vomica, green circles represent the targets of Nux vomica, and the size represents the degree.

The TCMSP database was used to search for the bioactive compounds of Nux vomica with the criteria of OB ≥ 30% and DL ≥ 0.18. The TCMSP database was also used to search for the targets of Nux vomica. As a result, 11 active compounds with the targets were found in Nux vomica. Previous studies have shown that strychnine is one of the major bioactive and principal toxic compounds of Nux vomica [9]. Therefore, strychnine was considered as a candidate in this study. The DL and pharmacokinetics of strychnine were subsequently evaluated using the online tool SwissADME. The results indicated that strychnine exhibited good gastrointestinal absorption and high DL properties, confirming that the strain was an active compound of Nux vomica. The targets of strychnine were predicted by SwissTargetPrediction, and the top 25 were viewed as key targets of strychnine. Collectively, we found 12 bioactive compounds and 72 targets in Nux vomica, and the details are shown in Tables 1 and 2. Subsequently, we constructed a Nux vomica bioactive compound-target network using Cytoscape (Figure 1).

### 3.2. GO and KEGG Pathway Enrichment Analysis of Nux Vomica

**Figure 2:**
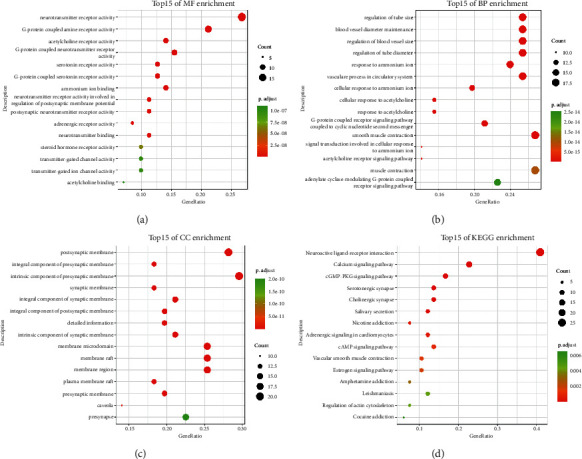
Gene Ontology and KEGG pathway enrichment analysis of 72 targets of Nux vomica bioactive compounds. (a) Representative bubble plots of molecular function (MF) of identified targets. (b) Representative bubble plots of biological processes (BP) of identified targets. (c) Representative bubble plots of cellular components (CC) of identified targets. (d) Representative bubble plots of KEGG pathway of identified targets. Gene ratio = count/set size.

To further investigate the 72 targets of the Nux vomica bioactive compounds, GO enrichment analysis was performed using biological processes (BP), molecular function (MF), and cellular component (CC) terms. A total of 103 MFs, 454 BPs, and 48 CCs were found to be enriched (*P* < 0.01). The GO analysis with the top 15 markedly enriched MF, BP, and CC terms is shown in Figures 2(a)–2(c). KEGG pathway enrichment analysis demonstrated that 72 targets of Nux vomica bioactive compounds were enriched in 17 associated pathways (*P* < 0.01). The top 15 KEGG pathways in which the targets of Nux vomica bioactive compounds were enriched are presented in Figure 2(d) and Table 3.

### 3.3. Target Genes of MG

Thirty-seven MG-related targets were obtained from the GENECARD database, and 42 were from the DisGeNET database. Seventy-nine disease target genes were retrieved after combining the results of both databases, and the duplicates were deleted. The details are listed in Table 4.

### 3.4. Nux Vomica-MG PPI Network

**Figure 3:**
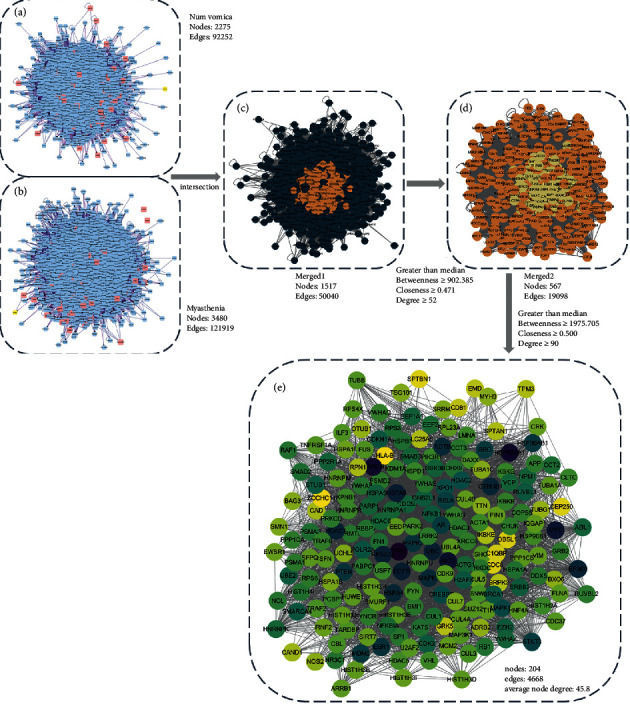
Identification of core targets for Nux vomica against MG. (a) The PPI network of Nux vomica-related targets (2,275 nodes and 92,252 edges). (b) The PPI network of MG-related targets (3,480 nodes and 121,919 edges). (c) Intersection of PPI networks (1,517 nodes and 5,0040 edges). (d) PPI network by the screening criteria of BC ≥ 902.385, CC ≥ 0.471, DC ≥ 52 (567 nodes and 19,098 edges). (e) Core-target PPI network by the screening criteria of BC ≥ 1985.705, CC ≥ 0.500, DC ≥ 90 (204 nodes and 4,668 edges). BC, betweenness centrality; CC, closeness centrality; DC, degree centrality.

The PPI networks of Nux vomica- and MG-related targets were constructed using Bisogenet. The PPI network of Nux vomica-related genes was constructed with 2,275 nodes and 92,252 edges (Figure 3(a)). In addition, the MG-related target network was constructed with 3,480 nodes and 121,919 edges (Figure 3(b)). Both networks were subsequently merged into an intersection with 1,517 nodes and 50,040 edges (Figure 3(c)). CytoNCA was used to assess the intersection of the PPI network by topological analysis. A Nux vomica on the myasthenia PPI network was first screened based on the criteria of “BC ≥ 902.385, CC ≥ 0.471, DC ≥ 52” (Figure 3(d)). A core-target PPI network was further screened using the criteria of “BC ≥ 1985.705, CC ≥ 0.500, DC ≥ 90,” which consists of 204 nodes and 4,668 edges (Figure 3(e)).

### 3.5. Enrichment Analysis of the Core-Target PPI Network

**Figure 4:**
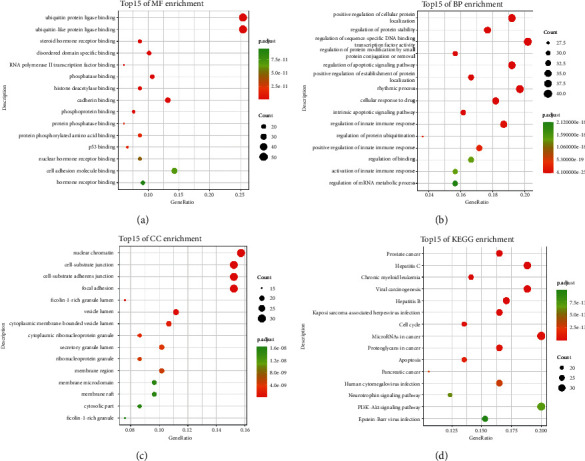
Gene Ontology and KEGG pathway enrichment analysis of 204 core targets for Nux vomica against MG. (a) Representative bubble plots of molecular function (MF) of core targets. (b) Representative bubble plots of biological processes (BP) of core targets. (c) Representative bubble plots of cellular components (CC) of core targets. (d) Representative bubble plots of KEGG pathway of core targets. Gene ratio = count/set size.

In the GO enrichment analysis, a total of 144 molecular functions, 1,570 biological processes, and 151 cell components were identified in 204 core targets of Nux vomica following MG treatment (*P* < 0.01). The top 15 significant GO terms, including MF, BP, and CC terms, are shown in Figures 4(a)–4(c). KEGG pathway enrichment analysis demonstrated that 132 pathways were enriched in 204 core targets (*P* < 0.01). The top 15 KEGG pathways enriched for the targets of bioactive compounds from Nux vomica are shown in Figure 4(d).

### 3.6. Enrichment Analysis of the Hub Genes

**Figure 5:**
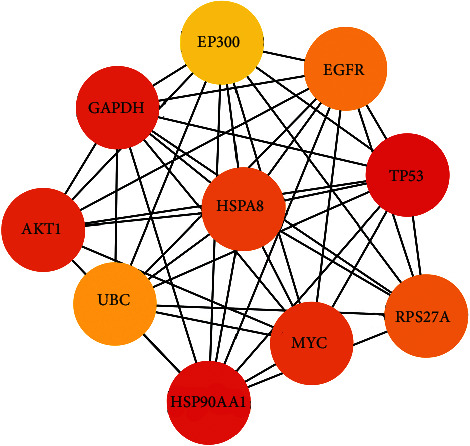
The PPI network of 10 hub genes.

Hub genes were selected using CytoHubba based on the core-target PPI network. The results showed that TP53, EGFR, UBC, MYC, HSP90AA1, EP300, HSPA8, RPS27A, AKT1, and GAPDH were the major hub nodes in the core-target PPI network, and the details are shown in Table 5. The PPI network of the hub genes is shown in Figure 5.

**Figure 6:**
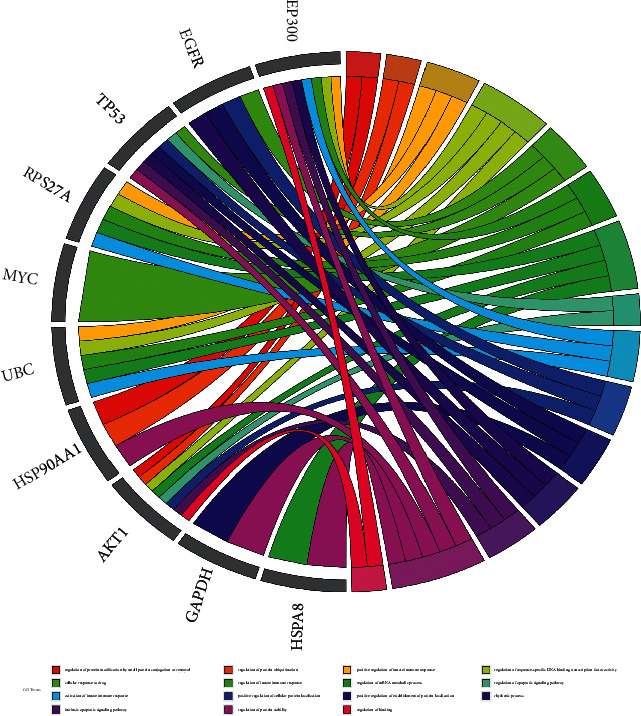
The top 10 GO terms of hub genes.

The results of the most representative GO terms included regulation of protein modification by small protein conjugation or removal, cellular response to drugs, activation of the innate immune response, intrinsic apoptotic signaling pathway, regulation of protein ubiquitination, regulation of the innate immune response, positive regulation of cellular protein localization, regulation of protein stability, positive regulation of the innate immune response, regulation of the mRNA metabolic process, positive regulation of establishment of protein localization, regulation of binding, regulation of sequence-specific DNA binding transcription factor activity, regulation of apoptotic signaling pathway, and rhythmic process (Figure 6).

**Figure 7:**
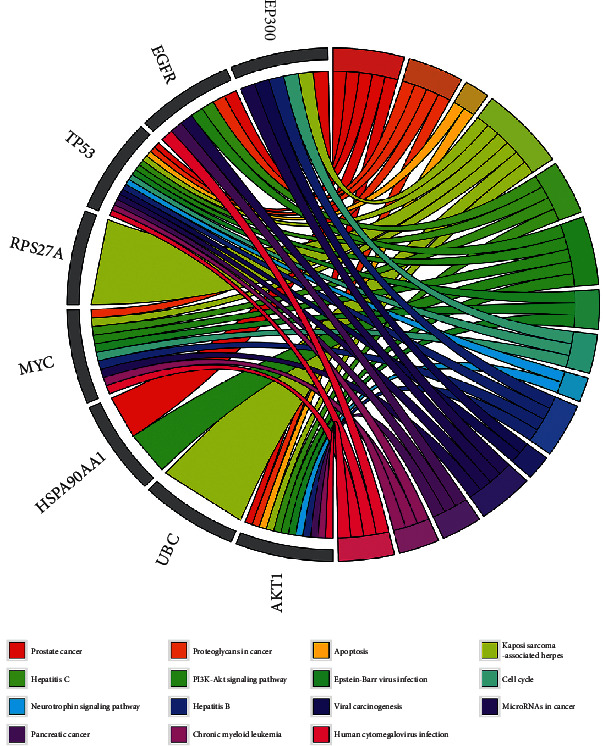
The top 15 pathways of hub genes.

In addition, the top 15 pathways in which the core targets were enriched were identified (Figure 7), mainly including the neurotrophin signaling pathway, PI3K-Akt signaling pathway, cell cycle, apoptosis, viral carcinogenesis, prostate cancer, hepatitis C, pancreatic cancer, Epstein-Barr (EB) virus infection, human cytomegalovirus infection, proteoglycans in cancer, hepatitis B, chronic myeloid leukemia, Kaposi sarcoma-associated herpes, and microRNAs (miRNAs) in cancer.

### 3.7. Molecular Docking Analysis

**Figure 8:**
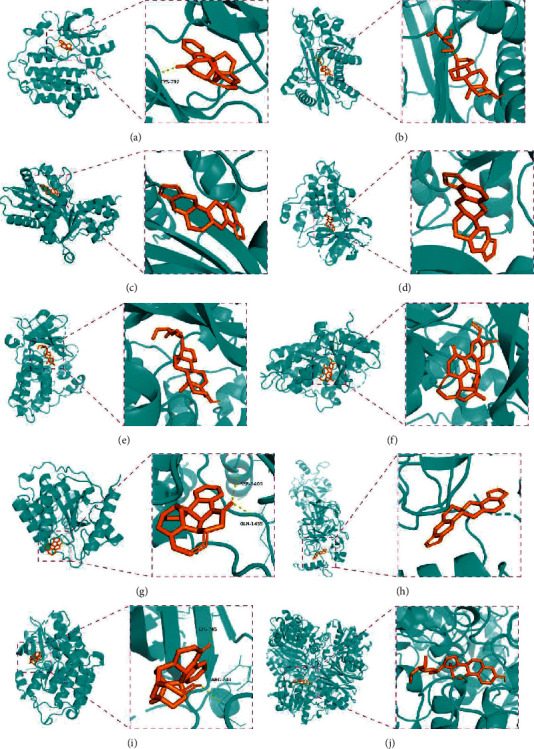
Molecular docking of hub genes with active compounds. (a) The binding poses of strychnine complexed with EGFR. (b) The binding poses of Stigmasterol complexed with EP300. (c) The binding poses of (S)-Stylopine complexed with EP300. (d) The binding poses of (S)-Stylopine complexed with EGFR. (e) The binding poses of Stigmasterol complexed with EGFR. (f) The binding poses of brucine-N-oxide complexed with EGFR. (g) The binding poses of strychnine complexed with EP300. (h) The binding poses of (S)-Stylopine complexed with TP53. (i) The binding poses of IsostrychnineN-oxide (II) complexed with EGFR. (j) The binding poses of Stigmasterol complexed with GAPDH.

The five bioactive compounds (strychnine, stigmasterol, (S)-stylopine, brucine-N-oxide, and isostrychnineN-oxide (II)) are shown in Table 6. The top ten hug targets (TP53, EGFR, UBC, MYC, HSP90AA1, EP300, HSPA8, RPS27A, AKT1, and GAPDH) were selected as the core compounds and core targets for molecular docking. AutoDock Vina assesses the binding of small molecules mainly by binding energy (affinity) to assess the binding strength of a small molecule to a protein. If the affinity is <0, the ligand can spontaneously bind to the receptor, and the smaller the value, the easier it is for the active ingredient to bind to the receptor. Therefore, the interaction between the active compound and its corresponding target was analyzed by the binding energy, and a threshold affinity <–5 kcal/mol was set in this study. A total of 50 docking pairs were obtained, as shown in Table 7.  Figure 8 shows the binding patterns of the top ten binding core targets and their corresponding compounds.

### 3.8. MG Mouse Model Establishment

**Figure 9:**
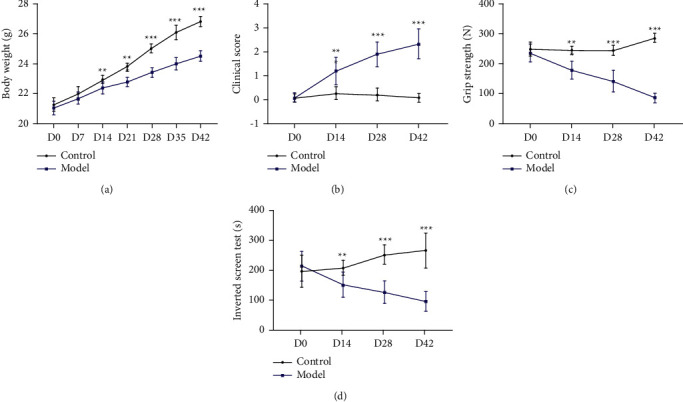
MG model constructs successful clinical examination indicators. (a) body weight; (b) clinical score; (c) grip strength; (d) inverted screen test.^*∗∗*^*P* < 0.01, and ^*∗∗∗*^*P* < 0.001*vs.* control group.

As shown in Figure 9, the body weight of the mice increased every week, and after 42 days, the body weight of the mice in the model group was significantly lower than that of the mice in the control group (*P* < 0.01). Clinical examinations were performed every 2 weeks. After 42 days, compared to the control group, the clinical scores were increased, the grip strengths were weakened, and the inverted screen times were shortened in the model group (*P* < 0.01).

### 3.9. Nux Vomica Improves Clinical Examination Indexes of MG Model Mice

**Figure 10:**
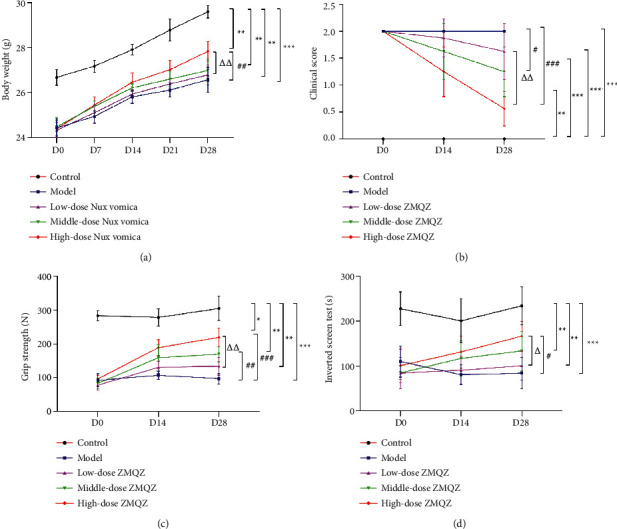
Nux vomica for MG clinical examination indicators. (a) Body weight; (b) clinical score; (c) grip strength; (d) inverted screen test. ^*∗*^*P* < 0.05, ^*∗∗*^*P* < 0.01, and ^*∗∗∗*^*P* < 0.001*vs*. control group; ^#^*P* < 0.05, ^##^*P* < 0.01, and ^###^^ ^*P* < 0.001*vs.* model group; ^Δ^*P* < 0.05, and ^ΔΔ^*P* < 0.01*vs.* low-dose Nux vomica group.

After treatment with Nux vomica, the clinical examination indicators of the MG model (weight, clinical score, grip strength, and inverted screen time) significantly improved. After 28 days of treatment, the weight of the MG mice increased significantly, clinical scores reduced, grip strength increased, and the inverted screen times were increased with the increased dose of Nux vomica (Figure 10).

### 3.10. Expression of Hub Targets

**Figure 11:**
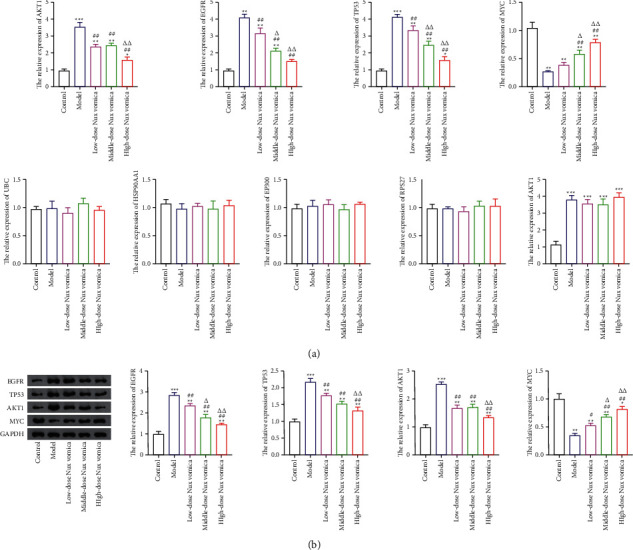
Hub targets expression. (a) The mRNA expression of EP300, EGFR, TP53, RPS27A, MYC, HSP90AA1, UBC, AKT1, GAPDH, and HSPA8 were detected by the qRT-PCR assay. (b) Western blot was used to detected the protein expression of MYC, EGFR, TP53, and AKT1 targets. ^*∗*^*P* < 0.05, ^*∗∗*^*P* < 0.01 and ^*∗∗∗*^*P* < 0.001 vs. control group; ^#^*P* < 0.05, and ^##^*P* < 0.01 vs. model group; ^ΔΔ^*P* < 0.01, and ^Δ^*P* < 0.05 vs. low-dose Nux vomica group.

To further verify the effect of Nux vomica against MG, we detected the expression of hub targets by qRT-PCR and western blotting. The results showed that compared to the model group, Nux vomica treatment significantly reduced EGFR and TP53 expression and increased MYC expression in a dose-dependent manner. Moreover, HSPA8 expression was not significantly changed, while AKT1 expression was significantly reduced, and the effect was particularly pronounced in the high-dose group. The qRT-PCR results also showed that the mRNA expression of RPS27, UBC, HSP90AA1, and EP300 was not significantly different between the groups (Figure 11).

## 4. Discussion

MG is an autoimmune disease of the neuromuscular junction, which is mainly mediated by acetylcholine receptor (AChR) antibody and muscle-specific kinase (MuSK) [27, 28]. Presently, traditional therapies for long-term MG treatment have several adverse effects in some populations. Therefore, it is vital to explore new methods for treating MG. Owing to their high efficiency and few side effects, TCMs have been widely used in MG treatment as an alternative or adjuvant therapy. In addition, a previous meta-analysis indicated that compared to using Western medication alone, TCMs could function as adjuvant therapies to improve the efficacy and decrease the side effects and relapse rate in treating MG [29, 30]. As a TCM, Nux vomica is warm in nature, with a slightly bitter flavor due to its various pharmacological activities. However, the effects and mechanism of action of Nux vomica on MG treatment remain unclear. Therefore, our study systematically explored the underlying mechanism of Nux vomica treatment for MG based on network pharmacology and further validated the therapeutic effect of Nux vomica on MG in combination with *in vivo* experiments.

Based on the public databases, 12 bioactive compounds of Nux vomica were collected by setting OB ≥ 30% and DL ≥ 0.18 (Table 1). Studies have isolated many chemical compounds from Nux vomica, including alkaloids, glycosides, steroids, and organic acids [9]. Among them, alkaloids, including strychnine, brucine-N-oxide, isobrucine, vomicine, and brucine N-oxide, are the primary chemical components of Nux vomica and have been reported to have anticancer effects. Additionally, strychnine has been shown to exhibit significant anti-inflammatory activity [31], while brucine-N-oxide possesses a strong antinociceptive potential [32]. Moreover, stigmasterol can inhibit the activity of acetylcholinesterase (AChE) and has antioxidant, anti-inflammatory, and neuroprotective effects [33–35]. Thus, many compounds from Nux vomica possess favorable biological activity, including inhibiting AChE and immunomodulation and may exert a mitigative effect on MG.

MG occurrence is closely related to continuous viral infections, genetic factors, and abnormal immune responses. Growing evidence has demonstrated that EBV infection is correlated with the occurrence of MG [36, 37]. For example, EBV infection could affect TLR7/9-dependent innate immune responses, which enhance the inflammatory response, B-cell dysfunction, and autoimmunity in the thymus of patients with MG [36, 37]. Moreover, a recent pilot study showed that hepatitis *E* infection was relevant to MG, at least in China, and may contribute to MG by inducing replication in the thymus, bystander activation, and stimulation of antigen-specific T cells [38]. Target identification is a critical step for the mechanism investigation on Nux vomica treating MG. According to the PPI network of active compound targets, Nux vomica has the characteristics of multiple components and targets in the treatment of MG. Top 10 potential targets (EP300, EGFR, TP53, RPS27A, MYC, HSP90AA1, UBC, AKT1, GAPDH, HSPA8) of active compounds from Nux vomica were predicted to be associated with MG treatment. These 10 hub targets were validated *in vivo*, and results showed that Nux vomica treatment downregulated the expression of EGFR, TP53, and AKT1, while upregulated the MYC level in MG model mice. These findings indicated that EGFR, TP53, AKT1, and MYC may be the key targets for Nux vomica treating MG. In addition, the pathways of Nux vomica against MG were related to EBV infection, hepatitis B, hepatitis C, and other viral infections based on KEGG analysis of the core genes. We also identified the miRNA pathway in cancer. An increasing number of studies have suggested that miRNAs are involved in the occurrence and development of MG [39]. The miRNA profile of peripheral blood from MG patients was sequenced using a next-generation sequencing approach, and 41 miRNAs were found to be differentially expressed in nonresponders versus responders to immunosuppressive therapies. More importantly, miR-181d-5p, -323b-3p, -340-3p, -409-3p, and -485-3p can modulate the immune response and drug metabolism processes in patients with MG [40]. In another study, miR-612, miR-3651, and miR-3653 were found to be highly expressed in the peripheral blood mononuclear cells of patients with AChR-positive early-onset MG (AChR-EOMG), suggesting that these miRNAs may be involved in the pathogenesis of AChR-EOMG [41]. It was predicted that the active ingredients of Nux vomica may regulate the immune response by regulating the miRNA signaling pathway, thereby playing a role in the treatment of MG.

## 5. Conclusions

Our study systematically analyzed the potential effects of Nux vomica in the treatment of MG based on network pharmacology and validated its effects in combination with *in vivo* experiments. The findings showed that the bioactive compounds of Nux vomica exerted therapeutic effects on MG by acting on multiple targets and multiple pathways. Based on the network pharmacology, this study confirmed the therapeutic effects of Nux vomica on MG probably via downregulating the expression of EGFR, TP53, and AKT1 and upregulating MYC level. Our data may provide a valuable theoretical basis for future MG diagnosis and treatment and also serve as a reference for further mechanistic research on Nux vomica treating MG. However, we also found that the mechanisms of Nux vomica against are involved in the PI3K-Akt signaling pathway and miRNAs, so further experiments are needed to prove these predictions. Besides, the therapeutic efficacy of Nux vomica on MG is needed to be verified in a clinical trial.

## Figures and Tables

**Table 1:** Bioactive Compounds of Nux vomica.

Chemical	Molecular Formula	Molecular Weight (g/mol)	OB (%)	DL
(+)-catechin	C_15_H_14_O_6_	290.27	54.83	0.24
(2R)-5, 7-dihydroxy-2-(4-hydroxyphenyl)chroman-4-one	C_15_H_12_O_5_	272.25	42.36	0.21
(S)-stylopine	C_19_H_17_NO_4_	323.37	51.15	0.85
brucine-N-oxide	C_23_H_26_N_2_O_5_	410.51	49.17	0.38
Icaride A	C_22_H_28_O_7_	404.5	48.74	0.43
Isobrucine	C_23_H_26_N_2_O_4_	394.5	33.58	0.8
Isostrychnine N-oxide (I)	C_21_H_25_N_2_O_3_	352.47	35.45	0.8
Isostrychnine N-oxide (II)	C_21_H_23_N_2_O_3_	350.45	37.33	0.8
Lokundjoside_qt	C_29_H_44_O_10_	552.65	32.82	0.76
Stigmasterol	C_29_H_48_O	412.77	43.83	0.76
Vomicine	C_22_H_24_N_2_O_4_	380.437	47.56	0.65
Strychnine	C_21_H_22_N_2_O_2_	334.4	High	YES

**Table 2:** The Target Genes of Nux vomica.

ID	Name
126	ADH1C
148	ADRA1A
147	ADRA1B
146	ADRA1D
150	ADRA2A
153	ADRB1
154	ADRB2
367	AR
847	CAT
1128	CHRM1
1129	CHRM2
1131	CHRM3
1132	CHRM4
1133	CHRM5
1135	CHRNA2
1504	CTRB1
2099	ESR1
2100	ESR2
3037	HAS2
3359	HTR3A
3757	KCNH2
4048	LTA4H
801	CALM1
4129	MAOB
4128	MAOA
3356	HTR2A
2556	GABRA3
1139	CHRNA7
3363	HTR7
2147	F2
2159	F10
5467	PPARD
5292	PIM1
3778	KCNMA1
8648	NCOA1
10499	NCOA2
4985	OPRD1
4988	OPRM1
5241	PGR
5328	PLAU
5644	PRSS1
5742	PTGS1
5743	PTGS2
6256	RXRA
6331	SCN5A
6530	SLC6A2
6531	SLC6A3
6532	SLC6A4
11255	HRH3
3741	KCNA5
1812	DRD1
1803	DPP4
3716	JAK1
1595	CYP51A1
1137	CHRNA4
2837	UTS2R
54878	DPP8
91039	DPP9
3352	HTR1D
1230	CCR1
9475	ROCK2
120892	LRRK2
3718	JAK3
3717	JAK2
1141	CHRNB2
6093	ROCK1
2155	F7
2554	GABRA1
4843	NOS2
4306	NR3C2
9475	ROCK2
1432	MAPK14

**Table 3:** The Top 15 KEGG Pathways of Nux vomica-Related Target Genes.

ID	Description	p.adjust	Count
hsa04080	Neuroactive ligand-receptor interaction	2.54*E* − 18	27
hsa04020	Calcium signaling pathway	3.70*E* − 09	15
hsa04022	cGMP-PKG signaling pathway	4.62*E* − 06	11
hsa04725	Cholinergic synapse	1.07*E* − 05	9
hsa04726	Serotonergic synapse	1.07*E* − 05	9
hsa04970	Salivary secretion	2.05*E* − 05	8
hsa05033	Nicotine addiction	0.000381	5
hsa04261	Adrenergic signaling in cardiomyocytes	0.000549	8
hsa04024	cAMP signaling pathway	0.001051	9
hsa04270	Vascular smooth muscle contraction	0.001575	7
hsa04915	Estrogen signaling pathway	0.001806	7
hsa05031	Amphetamine addiction	0.003161	5
hsa04810	Regulation of actin cytoskeleton	0.00453	8
hsa05140	Leishmaniasis	0.00453	5
hsa05030	Cocaine addiction	0.006586	4

**Table 4:** The Target Genes of MG.

ID	Name
1756	DMD
5376	PMP22
270	AMPD1
2992	GYG1
6904	TBCD
9499	MYOT
79823	CAMKMT
1437	CSF2
1440	CSF3
7273	TTN
6606	SMN1
1674	DES
6647	SOD1
7415	VCP
4000	LMNA
2110	ETFDH
2318	FLNC
64135	IFIH1
4621	MYH3
23345	SYNE1
2200	FBN1
773	CACNA1A
3123	HLA-DRB1
1292	COL6A2
55863	TMEM126B
55154	MSTO1
7157	TP53
1180	CLCN1
4982	TNFRSF11B
2218	FKTN
28976	ACAD9
8074	FGF23
178	AGL
4038	LRP4
1760	DMPK
4359	MPZ
26092	TOR1AIP1
3767	KCNJ11
57679	ALS2
1605	DAG1
4567	TRNL1
7276	TTR
5373	PMM2
9590	AKAP12
1134	CHRNA1
1146	CHRNG
4593	MUSK
5913	RAPSN
1145	CHRNE
43	ACHE
7124	TNF
1493	CTLA4
6261	RYR1
375790	AGRN
3586	IL10
3558	IL2
326	AIRE
3565	IL4
3458	IFNG
4155	MBP
10673	TNFSF13B
7112	TMPO
4049	LTA
940	CD28
941	CD80
246750	MYAS1
5354	PLP1
3106	HLA-B
7293	TNFRSF4
3115	HLA-DPB1
942	CD86
3119	HLA-DQB1
1604	CD55
1378	CR1
154	ADRB2
26191	PTPN22
959	CD40LG
285489	DOK7

**Table 5:** The Key Parameter Values of 10 Hub Genes.

Genes	Betweenness	Closeness	Degree	Eigenvector	LAC	Network
TP53	78488.11	0.592188	492	0.123322	39.82724	226.9392
EGFR	29951.12	0.552277	302	0.069005	26.71523	98.37902
UBC	20784.32	0.547688	278	0.07858	32.83094	97.09218
MYC	17003.02	0.539502	246	0.060742	22.79675	63.83153
HSP90AA1	12731.44	0.538544	245	0.075996	36.49796	85.84409
EP300	10000.75	0.532117	215	0.060767	34.1907	75.79397
HSPA8	7938.097	0.532303	200	0.065457	33.275	60.61926
RPS27 A	4139.276	0.51723	149	0.053001	29.77181	43.20848
AKT1	5381.846	0.51776	143	0.038709	21.11189	32.85155
GAPDH	2874.597	0.515646	111	0.042723	24.31532	29.43539

**Table 6:** The chemical structure of five active compounds.

Synonyms	Molecular Formula	2D Structure
Strychnine	C_21_H_22_N_2_O_2_	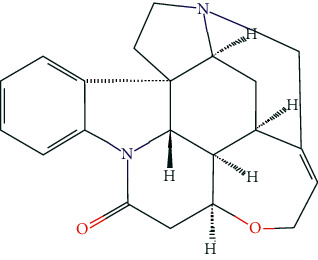

Stigmasterol	C_29_H_48_O	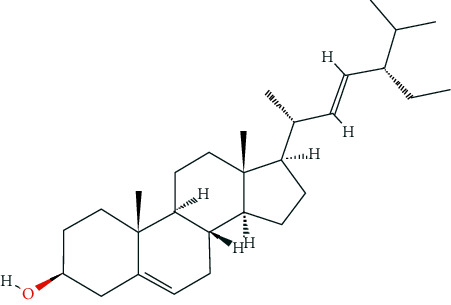

(S)-stylopine	C_19_H_17_NO_4_	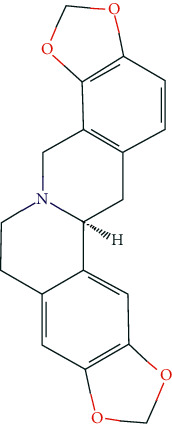

brucine-N-oxide	C_23_H_26_N_2_O_5_	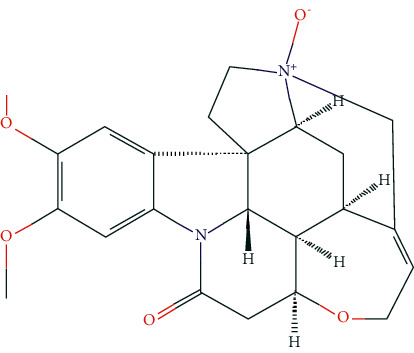

Isostrychnine N-oxide (II)	C_21_H_23_N_2_O_3_	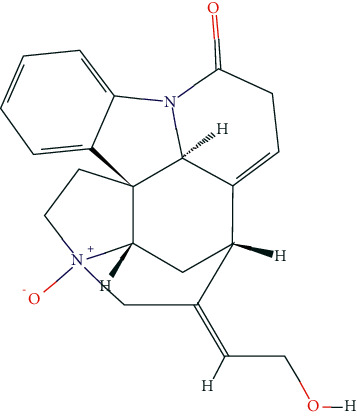

**Table 7:** The result of molecular docking.

Chem	PDB	Gene	Best affinity
Strychnine	3poz	EGFR	−9.9
Stigmasterol	3biy	EP300	−9.7
(S)-stylopine	3biy	EP300	−9.6
(S)-stylopine	3poz	EGFR	−9.6
Stigmasterol	3poz	EGFR	−9.5
brucine-N-oxide	3poz	EGFR	−9.5
Strychnine	3biy	EP300	−9.4
(S)-stylopine	1xqh	TP53	−9.4
IsostrychnineN-oxide (II)	3poz	EGFR	−9
Stigmasterol	1u8f	GAPDH	−9
(S)-stylopine	1u8f	GAPDH	−8.9
brucine-N-oxide	3ldq	HSPA8	−8.8
IsostrychnineN-oxide (II)	3biy	EP300	−8.7
brucine-N-oxide	1u8f	GAPDH	−8.7
IsostrychnineN-oxide (II)	1xqh	TP53	−8.6
IsostrychnineN-oxide (II)	1u8f	GAPDH	−8.6
Strychnine	1u8f	GAPDH	−8.6
Strychnine	1byq	HSP90AA1	−8.5
(S)-stylopine	3ldq	HSPA8	−8.4
IsostrychnineN-oxide (II)	1byq	HSP90AA1	−8.3
Strychnine	1xqh	TP53	−8.3
Strychnine	3ldq	HSPA8	−8.3
brucine-N-oxide	1byq	HSP90AA1	−8.3
Strychnine	5h07	UBC	−8.2
brucine-N-oxide	3biy	EP300	−8.2
(S)-stylopine	1byq	HSP90AA1	−8.2
IsostrychnineN-oxide (II)	5h07	UBC	−7.9
IsostrychnineN-oxide (II)	3ldq	HSPA8	−7.7
Stigmasterol	1xqh	TP53	−7.6
brucine-N-oxide	1xqh	TP53	−7.6
brucine-N-oxide	5h07	UBC	−7.5
Stigmasterol	1byq	HSP90AA1	−7.4
Stigmasterol	3ldq	HSPA8	−7.2
brucine-N-oxide	6sqs	RPS27A	−7.1
(S)-stylopine	5h07	UBC	−6.8
Stigmasterol	6sqs	RPS27A	−6.6
IsostrychnineN-oxide (II)	6sqs	RPS27A	−6.5
Stigmasterol	5h07	UBC	−6.5
(S)-stylopine	6sqs	RPS27A	−6.4
(S)-stylopine	1nkp	MYC	−6.4
Strychnine	6sqs	RPS27A	−6.3
brucine-N-oxide	1nkp	MYC	−6.3
Stigmasterol	1nkp	MYC	−6.1
brucine-N-oxide	1h10	AKT1	−5.9
IsostrychnineN-oxide (II)	1nkp	MYC	−5.8
Strychnine	1nkp	MYC	−5.7
(S)-stylopine	1h10	AKT1	−5.6
Strychnine	1h10	AKT1	−5.5
Stigmasterol	1h10	AKT1	−5.3
IsostrychnineN-oxide (II)	1h10	AKT1	−5.1

## Data Availability

The data used to support the findings of this study are available from the corresponding author upon request.

## Supplementary Materials

Supplementary Materials Table S1. The primer sequences for qRT-PCR.
