# Supplementary material for: Systems Pharmacology and Molecular Docking Reveals the Mechanisms of Nux Vomica for the Prevention of Myasthenia Gravis
Source: Evid Based Complement Alternat Med. 2022 Jun 26;2022:9043822. doi: 10.1155/2022/9043822 (PMC9251099; doi:10.1155/2022/9043822)
Supplement: Supplementary Materials — Table S1. The primer sequences for qRT-PCR. [file 9043822.f1.docx]

Table S1: The primer sequences for qRT-PCR.

| Gene | Sequence |
| --- | --- |
| AKT1 | F: 5’-CTGACTGAGTCCTACCCCTGA-3’ |
|  | R: 5’-TTCATGGTCACACGGTGCTT-3’ |
| EGFR | F: 5’-GTCAGAGATGCGACCCTCAG-3’ |
|  | R: 5’-TGTGCCTTGGCAGACTTTCT-3’ |
| TP53 | F: 5’-AAGTTAACCTGCCCACCTCG-3’ |
|  | R: 5’-CAACTGTACAGGGCACGTCT-3’ |
| MYC | F: 5’-ACAGTGTTCTCTGCCTCTGC-3’ |
|  | R: 5’-AAGTTCACGTTGAGGGGCAT-3’ |
| UBC | F: 5’-AGCCCAGTGTTACCACCAAGA-3’ |
|  | R: 5’-ACACCCAAGAACAAGCACAAG-3’ |
| HSP90AA1 | F: 5’-CCTGACGGACCCCAGTAAAC-3’ |
|  | R: 5’-TCCACAATGGTCAGGGTTCG-3’ |
| EP300 | F: 5’-TTGTGAAGAGCCCCATGGAT-3’ |
|  | R: 5’-GCCAGGGCTCTTGGTATTGT-3’ |
| RPS27 | F: 5’-TCCTTGGTCCAGGTTTCGAC-3’ |
|  | R: 5’-GACTACCGTTTGTGCATGGC-3’ |
| HSPA8 | F: 5’-TTGTGTGGTCTCGTCGTCAG-3’ |
|  | R: 5’-ACAGCGTTGGTAACGGTCTT-3’ |
| GAPDH | F: 5’-TCTCTGCTCCTCCCTGTTCC-3’ |
|  | R: 5’-ACTGTGCCGTTGAATTTGCC-3’ |
